# Genomic sequences of *Mycobacterium smegmatis* A cluster phages LBerry, Pembroke, and Zolita

**DOI:** 10.1128/mra.00504-24

**Published:** 2024-07-09

**Authors:** Nathan E. Berry, Marly S. Cassford, Colby J. Agostino, Ethan N. Dionne, Olivia J. Schmitt, Kristen A. Butela, Deborah Jacobs-Sera, Joseph A. DeGiorgis, Kathleen Cornely

**Affiliations:** 1 Department of Chemistry and Biochemistry, Providence College, Providence, Rhode Island, USA; 2 Department of Biological Sciences, University of Pittsburgh, Pittsburgh, Pennsylvania, USA; 3 Department of Biology, Providence College, Providence, Rhode Island, USA; 4 Whitman Center, Marine Biological Laboratory, Woods Hole, Massachusetts, USA; Department of Biology, Queens College, Queens, New York, USA

**Keywords:** bacteriophage, mycobacteria, genome analysis

## Abstract

LBerry, Pembroke, and Zolita are newly isolated bacteriophages that infect *Mycobacterium smegmatis* mc²155. Based on gene content similarity, LBerry and Pembroke are assigned to cluster A3, and Zolita is assigned to cluster A5. LBerry and Pembroke are 99% identical to Anaysia and Caviar, and Zolita is 99% identical to SydNat.

## ANNOUNCEMENT

The Mycobacterium genus of bacteria includes increasingly antibiotic-resistant human pathogens, such as *Mycobacterium tuberculosis* and *Mycobacterium abscessus* (1). With the increasing occurrence of antibiotic-resistant pathogens constituting a global threat to public health, phage therapy has recently been employed as an alternative treatment strategy. Some phages isolated using the nonpathogenic bacterial host *M. smegmatis* also infect pathogenic *Mycobacteria* and these phages can potentially be used in phage therapy (2, 3).

LBerry, Pembroke, and Zolita were isolated, purified, and their genomes were annotated through our participation in the SEA PHAGES program (4). Plaque purification, amplification, and production of high-titer lysates were performed as described in the Phage Discovery Guide (5).

**Fig 1:**
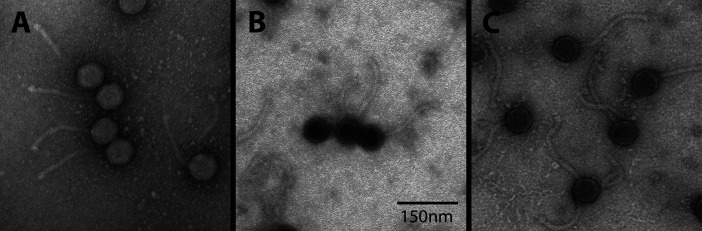
Images of (**A**) LBerry, (**B**) Pembroke, and (**C**) Zolita in negative-stained (1% uranyl acetate) taken by a JEOL 200 CX transmission electron microscope. All three phages have siphovirus morphology.

All three phages were isolated from damp grassy soil samples collected in the northeastern US; with LBerry isolated outside of a hotel, Pembroke from a former farm, and Zolita near a flower bed. Each sample was treated with 7H9 liquid medium, filtered (0.2 µm), and inoculated with *Mycobacterium smegmatis* mc^2^ 155. Samples were incubated at 37°C with shaking for 48 h, then plated on top agar with host bacteria to form plaques. Three rounds of purification were done for LBerry and Pembroke, and four rounds for Zolita. All three phages were determined to have siphovirus morphology via negative-strain transmission electron microscopy (Fig. 1). DNA from each phage was extracted from a high-titer lysate by phenol: chloroform: isoamyl: alcohol extraction (6) and sequenced by the Pittsburgh Bacteriophage Institute (Table 1). Raw reads were verified for accuracy using Consed v29.0 (7) and assembled using Newbler v2.9 (8). All phage genomes have a 3′ single-stranded overhang; the sequences are reported in Table 1 along with genome sizes and GC content for each phage. Based on gene sequence similarities, LBerry and Pembroke were assigned to the A3 cluster while Zolita was assigned to the A5 cluster (9, 10). LBerry and Pembroke are 99% identical to A3 cluster phages Anaysia OP021679 and Caviar ON970623 (11), and Zolita is 99% identical to A5 phage SydNat ON970625.

**TABLE 1:** Sequencing, genome, and phage characteristics

Parameter	LBerry	Pembroke	Zolita
Soil sample characteristics			
Collection date	17 October 2022	10 October 2022	29 August 2018
Collection location coordinates	43.058056 N 77.650556 W	42.076111 N 70.833056 W	41.843056 N 71.438611 W
Phage particle characteristics			
Capsid size (nm)	68–71 (*n* = 20)	59–63 (*n* = 20)	67–69 (*n* = 20)
Tail length (nm)	184–187 (*n* = 20)	189–192 (*n* = 20)	211–214 (*n* = 20)
Taxonomic identification			
Class	*Caudoviricetes*		
Genus	*Microwolfvirus*		*Benedictvirus*
Species	Unclassified		*Benedictvirus Zolita*
Sequencing			
Sequencing instrument	Illumina MiSeq v3 reagents		
Library prep kit	TruSeq DNA Nano Prep, S4 Flowcell, v1.5	NEB Ultra II Library Kit	
Number of reads	100,000	100,000	552,393
Length of reads (bp)	150-base single-end reads		
Shotgun coverage (×)	276	280	1,523
Phage genome characteristics			
Genome length (bp)	50,965	50,849	51,182
3′ single-stranded overhang sequence	CGGGTGGTAA	CGGGTGGTAA	CGGGAGGTAA
GC content (%)	64.0%	64.0%	60.9%

DNA Master v5.23.6 was used to perform the genome annotations (12). GeneMark v2.5 (13), Glimmer v3.02 (14), and Starterator v.546 (15) were used to determine gene starts. Protein functions were determined using HHpred (PDB, UniProt, Pfam-A v.36, and NCBI v.3.19 databases) (16, 17), BLASTp v.2.14.1 (18), and Phamerator (19). ARAGORN v.1.2.38 (20) and tRNAscan-SE v.2.0 (21) were used to identify tRNAs. Membrane proteins were predicted using TMHMM v.1.0.24 (22) and TOPCONS v.2.0 (23). Unless otherwise stated, default parameters were used for the programs listed.

Cluster A is the largest group of mycobacteriophages, with nearly 800 members. They are genetically diverse (24), and divided into 20 subclusters. LBerry, Pembroke, and Zolita follow the expected synteny of an A cluster phage beginning with a lysis cassette followed by structural proteins, integration proteins, replication/recombination proteins, an immunity repressor, and ending with a series of proteins of unknown function. The presence of immunity repressor and integrase genes in all three phages suggests that these phages could potentially adopt a temperate lifestyle (25). It has been determined that A3 cluster phages are able to infect *M. tuberculosis* H37Rv (26), indicating that LBerry and Pembroke could be further investigated for application in phage therapy.

## Data Availability

The genome sequence accession number for LBerry is OR725491 and the SRA accession number is SRX23702564. The genome accession number for Pembroke is OR725495 and the SRA accession number is SRX23702567. The genome accession number for Zolita is MN096372 and the SRA accession number is SRX18224444.
